# *In silico* approximation to aflatoxin B_1_ metabolism and sensitivity in commercial poultry species based on empirical mathematical equations

**DOI:** 10.1016/j.toxrep.2024.101752

**Published:** 2024-09-27

**Authors:** Hansen W. Murcia, Gonzalo Diaz, Rubén Darío Acosta

**Affiliations:** a Departamento de Biología, Facultad de Ciencias, Universidad Antonio Nariño, Carrera 3 este 47A – 15, Bogotá D.C., Colombia; b Laboratorio de Toxicología y Nutrición Aviar. Facultad de Medicina Veterinaria y Zootecnia, Universidad Nacional de Colombia, Bogotá D.C., Colombia; c Departamento de Sistemas Computacionales e Ingeniería Industrial, Facultad de Ingeniería, Universidad Nacional de Colombia, Bogotá D.C., Colombia

**Keywords:** Xenobiotic metabolism simulation, *in silico* simulation, Aflatoxin B_1_ metabolism, Cytotoxic pathway, Genotoxic pathway

## Abstract

Enzyme kinetic parameters for aflatoxin B_1_ metabolism have been reported for chicken, quail, turkey and duck, but an integrated *in silico* model has not been proposed. Both enzyme-catalyzed reactions and spontaneous reactions were modeled in the CellDesigner software and results were adjusted to Hill, Rational and Hoerl models. Results revealed that the higher amount of aflatoxin B_1_ epoxide produced in a short lapse of time and a low production of epoxide conjugated to glutathione explains the severe genotoxic effect of aflatoxin B_1_ in duck. Also, the higher amount of aflatoxicol produced is time-associated to aflatoxin B_1_ resistance in chicken. Finally, the cytotoxic effects in quail and duck are caused by a large aflatoxin B_1_ dialdehyde production in a short period of time.

## Highlights

• High AFB_1_-Gua production is related to a high production of AFBO in a short time.
• Poor AFB_1_-GSH production enhances AFBO adduction to DNA.
• Storing of AFB_1_ as AFL is related to resistance to AFB_1_ in chicken.
• Cytotoxic effects in quail and duck are related to a deficient AFB_1_ dialdehyde reduction.

## Graphical Abstract

**Figure:**
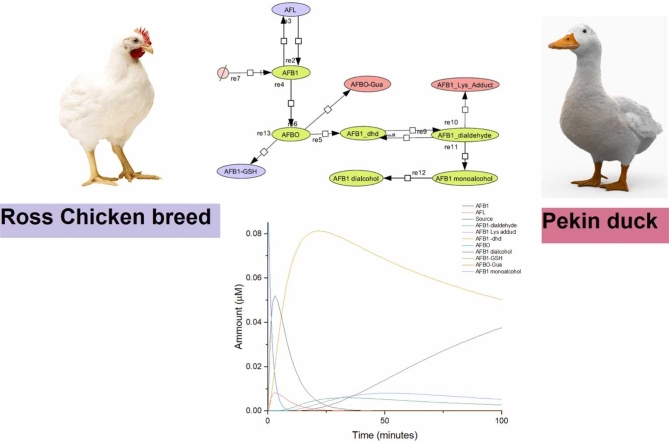

## 1 Introduction

Since the “X disease” outbreak in 1960, where thousands of turkey poults died because of the intake of a Brazilian peanut cake contaminated with high levels of aflatoxin B_1_ (AFB_1_; Blount, Turkey [6]), great advances have been achieved in the study of the metabolism of this mycotoxin. Large differences have been found in the adverse effects of AFB_1_ among commercial poultry species, being the duck the more sensitive, followed by quail>turkey>chicken [9]. Further, differences in the biotransformation rate of AFB_1_ into different products like the 8,9-dihydro-8-(*S*-glutathionyl)-9-hydroxy aflatoxin B_1_ (AFB_1_-GSH) have been found in poultry (Murcia *et al.,* 2021), where glutathione *S-*transferase (GST) is the enzyme that catalyzes the nucleophilic trapping of the bioactivated form of AFB_1_, the aflatoxin B_1_-8,9-epoxide (AFBO) with glutathione (GSH; [26]). Neutralization of AFBO restricts spontaneous adduction to guanine in DNA, preventing the production of the DNA-AFB_1_ adduct (AFB1-Gua; [18]) and consequently preventing genotoxicity. It is the case of rodents like rats and mice, where GST activity has a strong association with AFB_1_ resistance [18], [61]. In addition to GST enzyme activity, other enzyme kinetic parameters have been determined for poultry, as it was found for the aflatoxin B_1_ dihydrodiol production (AFB_1_-dhd), which is the hydrolyzed form of AFBO and in turn is able to rearrange into the AFB_1_ dialdehyde, producing spontaneous adducts with lysine in proteins causing cytotoxicity [15]. Moreover, enzyme kinetic parameters of the reduced form of AFB_1_ called aflatoxicol (AFL) have already been determined. The formation of AFL allows highly resistant birds, such as the chicken, to resist high AFB_1_ concentrations, by storing the mycotoxin in a non-toxic form such as AFL [43]. In the same way, enzyme kinetic parameters for aflatoxin B_1_ monoalcohol and AFB_1_ dialcohol already have been reported [44].

Integrated models of the metabolism of aflatoxin B_1_ in commercial poultry species have been proposed by Diaz and Murcia [16] and a kinetic model for human AFB_1_ metabolism with kinetic rates has been proposed by Guengerich et al. [25], but integration of the different enzyme kinetic parameters or kinetic rates in a simulation over time has not been performed in poultry, neither a non-linear model has been associated with each of the AFB_1_ biotransformation products. The use of New Approach Methodologies (NAMs) has been raised as a new trend, in order to avoid animal experiments and to assess the adverse effects of candidate xenobiotics [53]. According to this, the use of function models and the selection of a model that fit to data has become a fundamental scientific approach to find out the principles that explain a series of observations and to predict these observations [63] with no dependency on *in vivo* samples. Different function models have been proposed, and the choose of a model depends on the goodness-of-fit of the dataset to the selected model. For example, the Hill equation is a function model designed to adjust data that manifest a sigmoid behavior, as the binding of O_2_ to heamoglobin [27]. A modified version of this equation is presented by Gadagkar and Call [21], were a four-parameter logistic nonlinear regression model can be adjusted, where “C_m_” is the metabolite concentration at time X, “a” is the minimum asymptote or the response when time = 0, “b” is the maximum asymptote or the stabilized metabolite concentration for an infinite time, “c” is the time at which 50 % of the maximal concentration is reached and “d” is the slope at the steepest part of the curve (also known as the Hill slope). Eq. 1 presents the modified Hill equation.

(1) $$C_{m}=a+\frac{b-a}{1+{\left(\frac{c}{\mathrm{time}}\right)}^{d}}$$

In the other hand, Rational models are the ratio of two polynomial functions, that can take on an extremely wide range of shapes, accommodating to a much wider range of shapes than does the polynomial family, have better interpolatory and extrapolatory properties than polynomial models and are a particularly easy nonlinear models to fit (NIST/SEMATECH, 2023). Eq. 2 present a Rational model of the type linear/quadratic.

(2) $$C_{m}=\frac{a+b*\mathrm{time}}{1+c*\mathrm{time}+d*{\mathrm{time}}^{2}}$$

Another function model is the Hoerl function (Eq. 3). This model is part of the power law family, which are a group of equations that raises one or more parameters to the power of the independent variable and can be draw as convex or concave curves with or without inflection points or maxima/minima [30]. According to Wieczerzak et al. [63] the “a” parameter of the Hoerl model can be compared with that of the Gaussian model, representing the sensitivity, the impact or the effect of the system in consideration.

(3) $$C_{m}=ab^{\mathrm{time}}{\mathrm{time}}^{c}$$

In the case of those function models that produce Gaussian curves as the modified Hill equation or the Hoerl model, the area under the plot of concentration of reaction product versus time after dosage represents the extent of exposure to reaction products and their clearance rate from the body. By integrating over time, a more accurate estimate of the overall exposure is obtained [57]. On the other hand, the time of peak concentration (t_max_) of the reaction product shows the time course of drug concentration and the effect of the reaction product, such that the highest magnitude shows up at approximately the time of peak concentration [24].

Because an *in silico* simulation would allow to compare the production of these metabolites in a time-dependent manner and to associate this time-dependent metabolite behavior with poultry sensitivity, the present study aims at comparing the emulation of the time-dependent production of AFBO, AFL, AFB_1_-GSH, AFB_1_-dhd, AFB_1_ monoalcohol, and AFB_1_ dialcohol and to find the best-fitting models for each metabolite production reaction to finally associate differences in metabolite production with poultry species sensitivity to AFB_1_.

## 2 Materials and methods

**Fig. 1:**
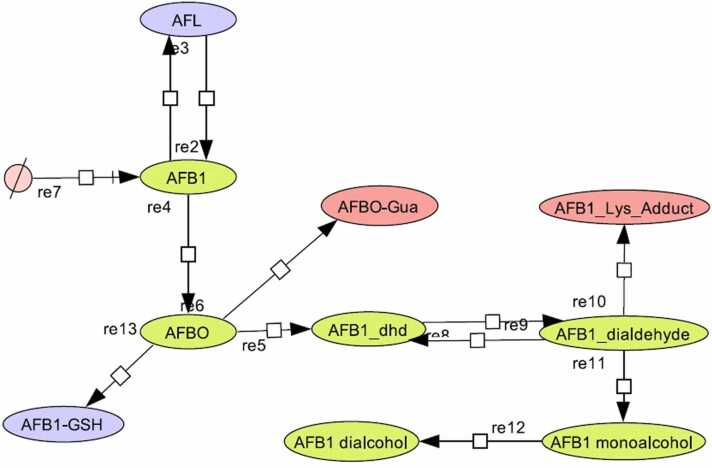
Aflatoxin B_1_ CellDesigner metabolism model for commercial poultry species.

An integrated model of AFB_1_ metabolism (Fig. 1) was generated for each of twelve individuals from five poultry commercial species, including two chicken breeds (n = 60) in the CellDesigner software version 4.4.2 [19], [20]. The integrated model was constructed with the biotransformation enzyme kinetic parameters K_m_ and V_max_ of the Michaelis-Menten model (v = V_max_[S]/K_m_ + [S]) obtained from Murcia and Diaz [42], [43], [44] and Diaz and Murcia [15], for Ross and Rhode Island Red (RIR) chicken breeds (*Gallus gallus ssp. domesticus*), Nicholas turkeys (*Meleagris gallopavo*), Japanese quails (*Coturnix Coturnix japonica*) and Pekin ducks (*Anas platyrhynchos ssp. domesticus*). Table 1 presents the average value ±standard deviation (SD) of these parameters by poultry species and by reaction. Reactions evaluated are as follows: AFB_1_ → AFBO reaction is driven by the cytochrome P450 (CYP) enzyme superfamily (E.C. number 1.14.–.-; [1]), specifically the CYP1A1 and CYP1A2 (E.C. number 1.14.1.1), the CYP2A6 (E.C. number 1.14.14.1) and the CYP3A4 (E.C. number 1.14.14.55, 1.14.14.56). Enzyme kinetic parameters for AFBO production are obtained indirectly from enzyme kinetic parameters obtained for AFB_1_-dhd [12], [13], [14], [15], [39], because AFBO is highly unstable in aqueous solutions (t_1/2_ = <1 s) and spontaneously the epoxide group in the AFBO is hydrolyzed, producing the AFB_1_ dihydrodiol [32]. AFB_1_ → AFL reaction is driven by an AFB_1_ cytosolic NADPH + H^+^ reductase and AFL → AFB_1_ reaction is driven by an AFL cytosolic dehydrogenase. AFB_1_ dialdehyde → AFB_1_ monoalcohol and AFB_1_ monoalcohol → AFB_1_ dialcohol reactions are driven by the aflatoxin B_1_ aldehyde reductase (AFAR; EC number 1.1.1.2) and the AFB_1_ → AFB_1_-GSH reaction is driven by a glutathione *S-*transferase (EC number 2.5.1.18).

**Table 1:** Average enzyme kinetic parameters V_max_ and K_m_ from different metabolic steps of AFB_1_ hepatic metabolism in commercial poultry species.

**Species**	**Reaction**	**V**_**max**_**(µM substrate/mg protein/minute)**	**SD**	**K_m_ (µM substrate)**	**SD**
Ross chickens	AFB_1_ → AFB_1_-dhd	23,0	7,8	131,8	26,2
	AFB_1_ → AFL	2,3	0,9	2,7	0,7
	AFL → AFB_1_	60,8	22,8	11,8	2,6
	AFB_1_ dialdehyde → AFB_1_ monoalcohol	8,6	4,5	80,2	46,5
	AFB_1_ dialdehyde → AFB_1_ dialcohol	1,3	0,7	19,4	11,6
	AFB_1_ → AFB_1_-GSH	0005	0001	65,6	14,4
RIR chickens	AFB_1_ → AFB_1_-dhd	44,8	5,9	112,5	33,4
	AFB_1_ → AFL	2,2	0,72	2,9	0,6
	AFL → AFB_1_	56,9	13,9	11,6	2,3
	AFB_1_ dialdehyde → AFB_1_ monoalcohol	40,2	22,0	393,2	227,0
	AFB_1_ dialdehyde → AFB_1_ dialcohol	1,2	0,66	21,6	14,4
	AFB_1_ → AFB_1_-GSH	0,0056	0,0005	47,4	7,1
Quail	AFB_1_ → AFB_1_-dhd	38,3	12,3	77,8	22,1
	AFB_1_ → AFL	2,0	1,1	5,6	2,5
	AFL → AFB_1_	92,8	31,6	29,8	6,8
	AFB_1_ dialdehyde → AFB_1_ monoalcohol	9,3	6,8	231,4	208,1
	AFB_1_ dialdehyde → AFB_1_ dialcohol	0,4	0,2	13,9	6,1
	AFB_1_ → AFB_1_-GSH	0003	0,001	92,6	25,2
Turkey	AFB_1_ → AFB_1_-dhd	23,4	8,3	49,3	7,6
	AFB_1_ → AFL	3,7	1,2	13,6	4,5
	AFL → AFB_1_	636,9	281,2	146,8	72,4
	AFB_1_ dialdehyde → AFB_1_ monoalcohol	10,5	6,1	72,8	45,9
	AFB_1_ dialdehyde → AFB_1_ dialcohol	1,5	0,7	12,6	6,1
	AFB_1_ → AFB_1_-GSH	0,0007	0,0003	87,6	24,5
Duck	AFB_1_ → AFB_1_-dhd	22,2	5,3	3,8	1,0
	AFB_1_ → AFL	11,8	3,1	46,8	7,7
	AFL → AFB_1_	762,7	666,5	84,0	16,5
	AFB_1_ dialdehyde → AFB_1_ monoalcohol	7,4	6,3	139,5	177,2
	AFB_1_ dialdehyde → AFB_1_ dialcohol	0,7	0,3	15,6	5,4
	AFB_1_ → AFB_1_-GSH	00013	0,0007	61,1	47,7

Reaction rates for non-enzymatically catalyzed reactions in the integrated model, as the AFBO adduction to guanine in DNA (*k* = 1.5 μM^−1^min^−1^), spontaneous hydrolysis of AFBO into AFB_1_-dhd (*k* = 42 min^−1^ + 0.126 μM^−1^min^−1^), rearrangement of AFB_1_-dhd into AFB_1_ dialdehyde forward (*k* = 0.12 μM^−1^min^−1^) and reverse (*k* = 0.012 min^−1^) reactions, and adduction of AFB_1_ dialdehyde with lysine (AFB1-Lys adduct; *k* = 2.4 ×10^−4^ μM^−1^min^−1^) were obtained from a scheme from Guengerich et al. [25], where *k* represents the reaction rate coefficient of the first-order reaction rate (rate = *k*[P]) in min^−1^ units, or the reaction rate coefficient of the second-order reaction rate (rate = *k*[P]^2^) in μM^−1^min^−1^ units, and P is the product concentration.

The simulation starts at the source (Fig. 1), where the initial concentration of AFB_1_ in serum plasma is 96 nM. This plasma AFB_1_ concentration is used according to estimates of maximum AFB_1_ serum plasma levels found in chicken after an oral administration dose of 2 mg/kg of body weight [36]. This serum plasma concentration used is lethal for duck (LD_50_ = 0.34 mg/kg BW) but not for turkey (LD_50_ = 3.2 mg/kg BW) or chicken (LD_50_ = 18.0 mg/kg BW; [9]). Transmembrane transport of AFB_1_ from plasma to hepatocyte cytosol (reaction re7 shown in the integrated model) is estimated to be 0.6 μM/mg cellular protein/minute and is independent of membrane carriers (simple diffusion [41]).

Simulation was run in the following software conditions: error tolerance = −6 and the solver chosen was SOSlib. In the species tab, the values for all chemical species were: compartment = default, quantity type = concentration, initial quantity = 0.000 except for the source = 0.096, boundary condition = false, constant = false. Parameters tab values were set according to the enzyme kinetic parameters for each individual, with units = substance and constant = true for all species. The simulation was run assuming an AFB_1_ single doses and a simulation time of <1440 minutes (1 day - acute exposure).

The dataset of “concentration vs time” obtained for the AFB_1_ and for the biotransformation products obtained from the integrated model per bird per poultry species in the CellDesigner software was then subjected to the CurveExpert Professional Software version 2.7.3 [30], to search in all available regressions for the function model with the lowess smoothing and the best fit to data. The criterion for selection of the function model was the score value obtained after data fitting to the set of models supplied by the software (the highest score value) and the goodness-of-fit of the data to the function model represented by the coefficient of determination (R^2^ [40]). The R^2^ value was calculated for function model and poultry species. After function model selection, the function model parameters were determined by non-linear regression using the Marquardt method. In the same way, the “time to peak” was determined by the ordinary differential equation (ODE) of the Hoerl and Rational function models (supplementary material) and the Area Under the Curve (AUC) was determined by the numerical method, implementing a Romberg-type integration scheme (numerical integration method that uses extrapolation of trapezoidal sums to approximate an integral over a domain) under a QUAD subroutine [55], by integrating the area of the model function in the time range of 0–400 minutes for AFB_1_ dialdehyde production, 0–500 minutes for AFB_1_ monoalcohol production, 0 – 15 minutes for AFBO production and 0 – 20 minutes for AFL production. Normal distribution of residuals was tested by the Shapiro-Wilk test, homogeneous variance with a Leven’s test, and residual independence was performed with a “residual versus value” graph [2]. Inter-species differences in Hill, Hoerl, or Rational model parameters were determined by using the ANOVA test and multiple comparisons were made by a Tukey test. All analyses were performed using the Statistical Analysis System software [55].

## 3 Results

According to the highest score and the goodness-of-fit for the “C_m_ vs time” dataset obtained from AFB_1_-Gua, AFB_1_-GSH, AFB_1_-lysine, and AFB_1_ dialcohol products, the function model with the best adjustment was the modified Hill equation (Eq. 1) and the parameters determination is shown in Table 2. For the dataset obtained from AFB_1_ dialdehyde and AFB_1_ monoalcohol products, the best model was the Rational model (Eq. 2), and the parameters determination is presented in Table 3. In the case of the dataset obtained from AFBO and AFL products, the model with the best score was the Hoerl model (Eq. 3), and model parameters are presented in Table 4. After function model selection, results are presented by reaction step as follows.

**Table 2:** Comparison of model parameters obtained by non-linear regression of AFB_1_-Gua, AFB_1_-GSH, AFB_1_-Lys, and AFB_1_ dialcohol (modified Hill model). R^2^: coefficient of determination; a: minimum asymptote or the response when time = 0; b: maximum asymptote or the stabilized metabolite concentration; c: time at which 50 % of the maximal concentration is reached; d: slope at the steepest part of the curve. All values are presented as the mean of 12 individuals ± standard deviation. Values with different letters are statistically significant.

Reaction product	Poultry species	Model parameters								
		a		b		c		d		R^2^
AFB_1_-Gua	Duck	c	−0.0039 ± 0.003	a	2.1 ± 0.2	c	0.9 ± 0.1	c	2.2 ± 0.18	0.9997
	RIR	ab	0.0028 ± 0.001	b	1.0 ± 0.1	b	2.9 ± 0.3	a	2.7 ± 0.07	0.9998
	Quail	a	0.0035 ± 0.002	b	1.0± 0.3	b	2.7 ± 0.7	a	2.7 ± 0.10	0.9997
	Ross	b	−0.0002 ± 0.001	c	0.5± 0.2	a	5.1 ± 1.5	b	2.3 ± 0.20	0.9999
	Turkey	a	0.0035 ± 0.002	b	1.0 ± 0.2	b	2.7 ± 0.4	a	2.7 ± 0.04	0.9997
AFB_1_-GSH	Duck	bc	1.6E−04 ± 1.2E−04	b	0.09 ± 0.07	c	1.4 ± 0.1	c	1.7 ± 0.1	0.9996
	RIR	a	8.0E−04 ± 4.6E−04	a	0.35 ± 0.23	b	3.9 ± 0.7	a	2.0 ± 0.2	0.9998
	Quail	ab	3.4E−04 ± 2.7E−04	b	0.09 ± 0.01	b	3.7 ± 1.0	ab	2.0 ± 0.1	0.9998
	Ross	c	−2.3E−04 ± 1.9E−04	ab	0.18 ± 0.03	a	7.7 ± 2.5	bc	1.7 ± 0.1	0.9999
	Turkey	bc	8.6E−05 ± 4.8E−05	b	0.02 ± 0.01	b	3.6 ± 0.5	ab	2.0 ± 0.1	0.9998
AFB_1_-Lys	Duck	a	−0.02 ± 0.010	ab	1.6 ± 1.2	b	50.7 ± 9.9	c	2.0 ± 0.1	0.9998
	RIR	a	−0.02 ± 0.003	bc	0.6 ± 1.7	bc	46.0 ± 3.2	bc	2.1 ± 0.1	0.9995
	Quail	a	−0.04 ± 0.012	a	2.8 ± 1.0	a	64.6 ± 7.2	ab	2.2 ± 0.1	0.9998
	Ross	a	−0.01 ± 0.005	bc	0.6 ± 0.3	bc	54.2 ± 8.7	a	2.4 ± 0.2	0.9990
	Turkey	a	−0.01 ± 0.003	c	0.3 ± 0.1	c	40.6 ± 2.7	bc	2.0 ± 0.1	0.9994
AFB_1_ dialcohol	Duck	ab	−3.5 ± 1.2	bc	89.9 ± 0.9	b	136.0 ± 20.6	ab	1.5 ± 0.1	0.9995
	RIR	b	−4.7 ± 0.9	b	90.2 ± 0.7	bc	117.4 ± 10.1	bc	1.5 ± 0.1	0.9992
	Quail	a	−2.5 ± 0.7	c	88.6 ± 0.4	a	165.5 ± 18.3	a	1.7 ± 0.1	0.9995
	Ross	bc	−4.8 ± 1.3	b	89.7 ± 1.1	c	116.8 ± 13.7	bc	1.5 ± 0.1	0.9989
	Turkey	c	−6.9 ± 0.6	a	92.1 ± 0.7	c	98.3 ± 4.2	c	1.3 ± 0.1	0.9994

**Table 3:** Comparison of model parameters obtained by non-linear regression of AFB_1_ dialdehyde, and AFB_1_ monoalcohol (Rational model). t_max_: time to peak; C_max_: concentration at t_max_; AUC: area under the curve; R^2^: coefficient of determination. All values are presented as the mean of 12 individuals ± standard deviation. Values with different letters are statistically significant.

Reaction product	Poultry species	Model parameters																	
		a		b		c		d		t_max_ (minutes)			C_max_ (pM)			AUC (pmol.minute/L)			R^2^
AFB_1_ dialdehyde	Duck	a	−0.2 ± 0.02	a	0.5 ± 0.04	a	−0.02 ± 0.003	ab	0.0016 ± 0.0007		b	27.2 ± 5.6		a	9.0 ± 2.5		b	1201.5 ± 501.3	0.9943
	RIR	c	−0.5 ± 0.05	c	0.3 ± 0.03	b	−0.03 ± 0.002	ab	0.0015 ± 0.0003		b	27.3 ± 2.4		b	6.6 ± 0.8		c	748.8 ± 115.8	0.9933
	Quail	d	−0.7 ± 0.14	b	0.4 ± 0.07	a	−0.02 ± 0.012	c	0.0008 ± 0.0002		a	37.6 ± 4.4		a	11.1 ± 2.6		a	1721.1 ± 376.7	0.9925
	Ross	b	−0.4 ± 0.17	d	0.2 ± 0.06	b	−0.03 ± 0.004	c	0.0010 ± 0.0004		a	35.2 ± 7.3		b	5.9 ± 1.6		c	714.6 ± 231.4	0.9936
	Turkey	bc	−0.4 ± 0.06	c	0.3 ± 0.05	b	−0.03± 0.003	a	0.0020 ± 0.0004		b	23.4 ± 2.0		b	5.1 ± 0.9		c	534.2 ± 119.9	0.9924
AFB_1_ monoalcohol	Duck	a	−0.3 ± 0.01	a	0.2 ± 0.05	a	−0.02 ± 0.004	bc	0.0004 ± 0.0002		b	56.3 ± 11.7		ab	11.5± 3.9		ab	2071.0 ± 1004.4	0.9917
	RIR	c	−0.5 ± 0.09	ab	0.2 ± 0.03	b	−0.03 ± 0.003	b	0.0005 ± 0.0001		b	48.6± 7.0		bc	9.8 ± 2.6		bc	1496.6 ± 523.0	0.9930
	Quail	c	−0.5 ± 0.09	bc	0.2 ± 0.03	a	−0.02 ± 0.003	c	0.0002 ± 0.0001		a	76.2 ± 10.3		a	13.4 ± 3.0		a	2749.9 ± 856.8	0.9896
	Ross	c	−0.5 ± 0.12	c	0.1 ± 0.03	b	−0.03 ± 0.005	b	0.0004 ± 0.0002		b	52.9 ± 10.2		cd	8.1 ± 2.9		cd	1213.8 ± 559.1	0.9934
	Turkey	b	−0.4 ± 0.05	bc	0.2 ± 0.02	c	−0.03 ± 0.002	a	0.0009 ± 0.0002		c	34.6 ± 3.3		d	5.5 ± 1.0		d	676.7 ± 145.1	0.9934

**Table 4:** Comparison of model parameters obtained by non-linear regression of AFBO, and AFL (Hoerl model). t_max_: time to peak; C_max_: concentration at t_max_; AUC: area under the curve; R^2^: coefficient of determination. All values are presented as the mean of 12 individuals ± standard deviation. Values with different letters are statistically significant.

Reaction product	Poultry species	Model parameters																
		a		b		c			t_max_ (minutes)			C_max_ (pM)			AUC (pmol.minute/L)			R^2^
AFBO	Duck	a	2977.3 ± 211.8	c	0.3 ± 0.02		c	0.7 ± 0.13		c	0.6 ± 0.1		a	1032.7 ± 58.7		a	2059.8 ± 66.2	0.9889
	RIR	b	482.5 ± 106.6	b	0.6 ± 0.04		a	1.0 ± 0.02		b	2.2 ± 0.2		b	380.5 ± 45.7		a	2240.0 ± 31.3	0.9989
	Quail	b	670.4 ± 394.3	b	0.6 ± 0.09		a	1.0 ± 0.04		b	2.0 ± 0.5		b	435.9 ± 125.5		a	2236.0 ± 45.0	0.9979
	Ross	c	191.2 ± 84.6	a	0.8 ± 0.06		b	0.9 ± 0.06		a	3.8 ± 1.2		c	232.1 ± 83.6		a	2220.7 ± 1205.0	0.9939
	Turkey	b	597.1 ± 206.4	b	0.6 ± 0.05		a	1.0 ± 0.02		b	2.0 ± 0.3		b	423.5 ± 67.9		a	2255.9 ± 14.6	0.9992
AFL	Duck	d	1.3 ± 1.1	c	0.3 ± 0.02		bc	1.2 ± 0.3		c	0.9 ± 0.2		d	0.4 ± 0.3		d	0.8 ± 0.7	0.992
	RIR	a	6.6 ± 0.8	b	0.6 ± 0.04		ab	1.3 ± 0.1		b	2.5 ± 0.3		b	6.0 ± 0.8		b	33.5 ± 9.6	0.995
	Quail	bc	4.5 ± 1.1	b	0.6 ± 0.09		a	1.5 ± 0.2		b	2.6 ± 0.6		c	4.0 ± 0.9		bc	22.3 ± 10.7	0.996
	Ross	ab	5.6 ± 1.2	a	0.8 ± 0.07		c	1.1 ± 0.1		a	4.0 ± 0.9		a	8.2 ± 2.5		a	85.8 ± 42.8	0.984
	Turkey	cd	3.0 ± 1.0	b	0.6 ± 0.05		ab	1.4 ± 0.1		b	2.4 ± 0.3		c	2.6 ± 1.1		cd	14.1 ± 6.7	0.996

### 3.1 AFBO production

**Fig. 2:**
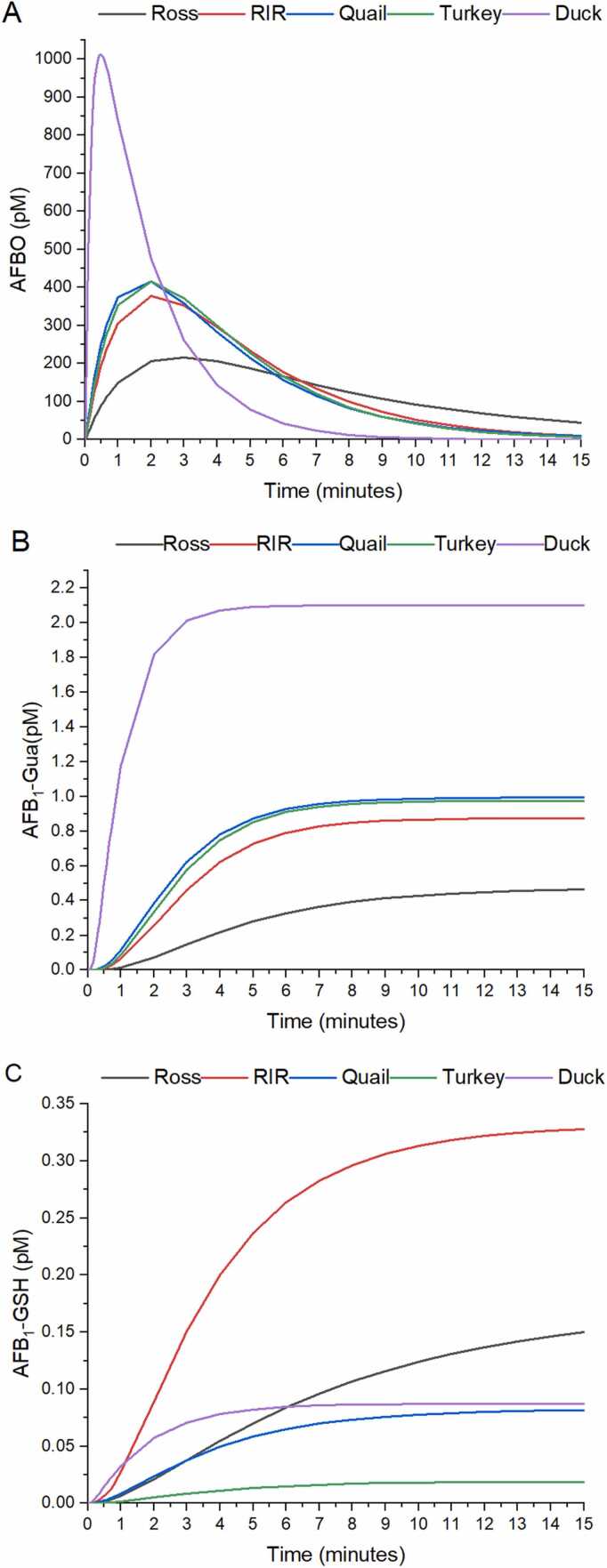
Average production of AFBO (A), AFB_1_-Gua (B), and AFB_1_-GSH (C) over a lapse of 15 minutes, for 12 individuals from 5 commercial poultry species. RIR: Rhode Island Red breed.

Fig. 2A shows the change in AFBO concentration (AFB_1_ epoxidation activity). During the first 5 minutes, an AFBO peak appears and fades out until zero after 15 minutes for all poultry species. The highest concentration reached was that of the duck (1032.6 ± 58.7 pM) and at the shortest time (0.6 ± 0.1 minutes). In contrast, the lowest peak was found for the Ross chicken breed (232.1 ± 83.6 pM; 4.5 times lower) which also occurred at a later time (3.8 ± 1.2 min; Table 4). In all cases the AUC did not present statistical differences.

### 3.2 AFB_1_-Gua production and AFB_1_-GSH production

The adduction of AFBO with DNA (AFB_1_-Gua production) is shown in Fig. 2B. Production of the adduct reaches a maximum of 2.1 ± 0.2 pM in the duck (value of the b model parameter), while in the Ross chicken breed it reaches a maximum at 0.5 ± 0.2 pM (3.8 times lower than the duck; Table 2). It is interesting to note that the duck reaches the maximum AFB_1_-Gua production much faster than the Ross breed. Regarding AFB_1_-GSH production (Fig. 2C), the RIR chicken breed has the highest AFB_1_-GSH production at 0.4 ± 0.2 pM (value of the b model parameter), after 15 minutes of simulation. In the duck, AFB_1_-GSH production reaches a maximum concentration of 0.1 ± 0.1 pM (3.9 times lower than the RIR breed) and in the turkey, AFB_1_-GSH production reaches a maximum of 0.02 pM (16.7 times lower compared to the RIR breed). AFBO and AFB_1_-GSH production showed statistical differences (p <0.05) among the different poultry species (see Table 2).

### 3.3 Net AFL production

**Fig. 3:**
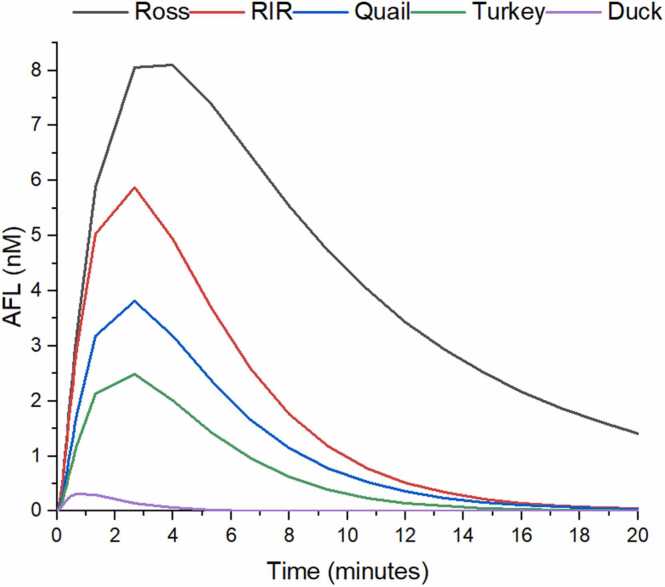
Average production of AFL over a lapse of 20 minutes, for 12 individuals from 5 commercial poultry species. RIR: Rhode Island Red breed.

AFL production encompasses two enzyme-catalyzed activities: AFB_1_ reductase and AFL oxidoreductase. For this reason, a change in AFL concentration (in nM) affects the net production of AFL. Fig. 3 shows the appearance of a peak that reached a maximum between 3 and 5 minutes of simulation and fades away to 1 nM between 10 and 20 minutes. Peak height ranged from 8.2 ± 2.5 pM in the Ross chicken breed to 0.4 ± 0.3 pM in duck (23.5 times lower than Ross; Table 4). Regarding the AUC, the Ross breed had the higher value by far, followed by the RIR breed > quail > turkey > duck (Table 4).

### 3.4 AFB_1_ dialdehyde production and production of AFB_1_- Lys

**Fig. 4:**
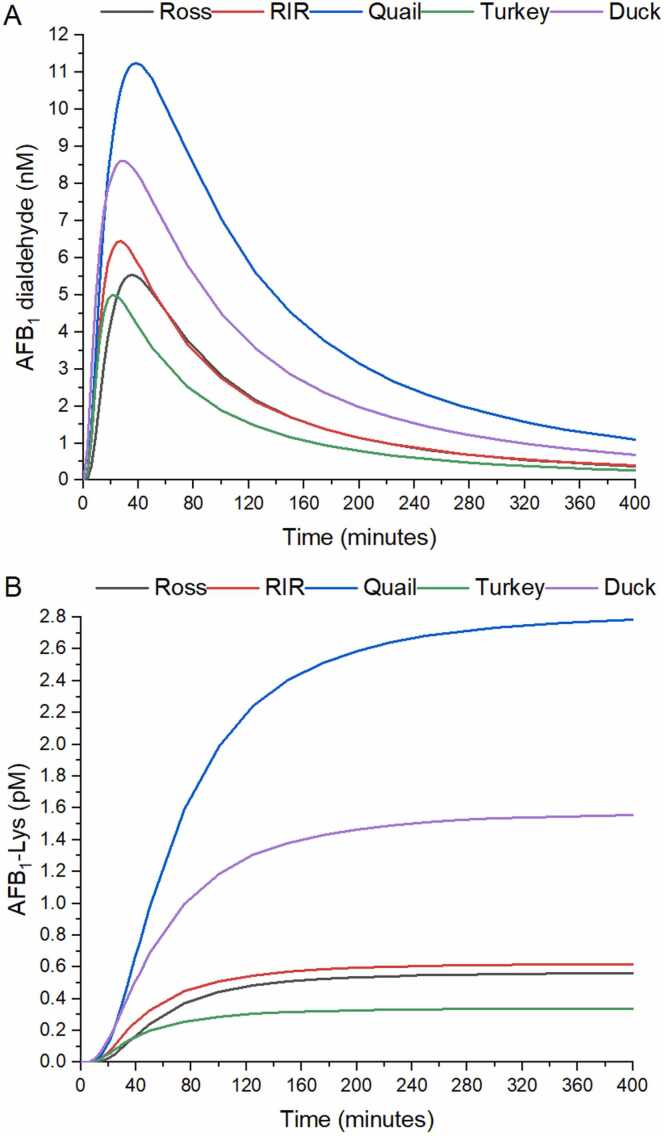
Average production of AFB_1_ dialdehyde (A), and AFB_1_-Lys (B) over a lapse of 400 minutes, for 12 individuals from 5 commercial poultry species. RIR: Rhode Island Red breed.

Fig. 4A presents the change in AFB_1_ dialdehyde concentration. Between 30 and 50 minutes of simulation AFB_1_ dialdehyde reaches a maximum in all species and then, fades out to less than 2 nM after 400 minutes. The quail and the duck showed the higher AFB_1_ dialdehyde peak (11.1 ± 2.6 and 9.0 ± 2.5 nM, respectively) and the largest AUC values (1721.1 ± 376.7 and 1201.5 ± 501.3 nmol*minute/L, respectively) with a magnitude more than two times higher in quail compared to the RIR breed (6.6 ± 0.8 pM), Ross breed (5.9 ± 1.6 pM), or Turkey (5.1 ± 0.9 pM; Table 3). Fig. 4B presents the production of AFB_1_-Lys adducts approaching to a plateau concentration after 400 minutes in all poultry species. Quail and duck reached the highest plateau with values of 2.8 ± 1.0 and 1.6 ± 1.2 pM, respectively. Turkey presented the lowest AFB_1_-Lys plateau concentration of 0.3 ± 0.1 pM (Table 2).

### 3.5 Production of AFB_1_ monoalcohol and AFB_1_ dialcohol

**Fig. 5:**
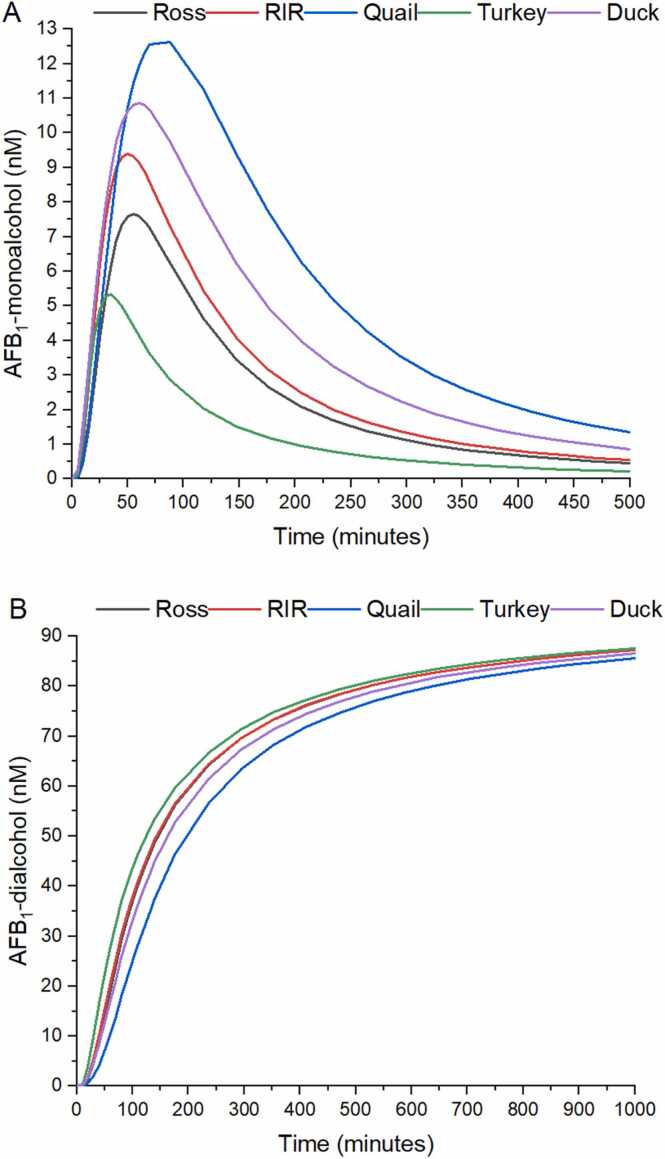
Average production of AFB_1_ monoalcohol (A), and AFB_1_ dialcohol (B) over a lapse of 500 and 1000 minutes, respectively, for 12 individuals from 5 commercial poultry species. RIR: Rhode Island Red breed.

Fig. 5A shows AFB_1_ monoalcohol production and Fig. 5B presents the AFB_1_ dialcohol production. AFB_1_ monoalcohol production reaches a maximum before 100 minutes, and AFB_1_ dialcohol production increases further than 1440 minutes in all poultry species. The highest AFB_1_ monoalcohol maximum peak reaches a value of 13.4 ± 3.0 pM in quail, and the lower was present in turkey (5.5 ± 1.0 pM). Similarly, the largest AUC value was the one recorded for quail (2749.9 ± 856.8 pmol/minute/L) and the lowest the one of the turkey (676.7 ± 145.1 pmol/minute/L. AFB_1_ dialcohol plateau values ranged between 88.6 ± 0.4 (for the quail) and 92.08 ± 0.7 nM (for the turkey; Table 2).

## 4 Discussion

The different toxic effects of AFB_1_ in poultry can be explain by clustering those reactions that produce toxic products (for example AFBO or AFB_1_ dialdehyde) and those reactions that inactivates these toxic products (for example AFB_1_-GSH or AFB_1_ dialcohol) into two pathways: the genotoxic and the cytotoxic pathways.

The “genotoxic pathway” starts with the comparison of AUC among the different poultry species evaluated, resulting in a statistically equal amount of AFBO produced by all species; however, there are large differences in the time to reach the peak. The Ross chicken breed is the species that reaches a maximum of AFBO in a longer time which is reflected on the maximum concentration (232.1 ± 83.6 pM). The Ross breed is followed by the RIR chicken breed, the quail, and the turkey, which present an intermediate t_max_ and C_max_. Finally, the duck presents the highest AFBO concentration peak (1032.7 ± 58.7 pm), which was more than 4 times higher than the Ross breed value, in a shorter time (0.6 ± 0.1 minutes), around 6 times shorter than the Ross breed. At this point, the exposure to AFBO was much higher for the duck, resulting theoretically in a higher attack of AFBO to DNA, leading to a higher amount of DNA adducts and DNA damage. Considering the AFB_1_-Gua production, it is important to note that the duck species present the highest AFB_1_-Gua peak production, which suggest a largest DNA damage. The duck produces 2.1 ± 0.2 pM of AFB_1_-Gua compared to Ross Breed, which only produces 0.5 ± 0.2 pM of AFB_1_-Gua. Fig. 2A and 2B show how the production of AFB_1_-Gua increases as AFBO production rises. In addition to the deleterious effects of AFB_1_, the effect of glutathione *S*-transferase (GST) activity seems to be partially related to sensitivity. The highest value of AFB_1_-GSH production corresponded to chicken breeds (Ross and RIR), the lower values to mid-tolerant species (quail and turkey) and an intermediate value to the most sensitive species (duck). The time needed to reach half the maximum of AFB_1_-GSH amount is the longest in the Ross breed and the shortest in the duck, suggesting that duck detoxification of AFB_1_ through GST activity tries to inactivate the AFBO produced by CYP450 enzymes. Despite this, the enzyme capacity is overwhelmed by the high AFBO-level production. In the same way, the high capacity of GST activity in chicken breeds and the low AFBO production explain why half the maximum production of AFB_1_-GSH in this poultry species is accomplished at longer times. Thus, the origin of the high rate of DNA adduction by AFB_1_ in the duck is the massive production of AFBO through CYP450 enzymes and the low GST activity. There is no evidence of the development of hepatocarcinoma due to a single dose of AFB_1_ in duck [58], which in turn suggest that continuous administration of AFB_1_ is required to develop tumors.

Regarding AFL production, chicken and quail are recognized as being resistant to the adverse effects of AFB_1_. Results demonstrate that there is a very large difference in AFL production between the Ross breed and the duck, being around 106 times higher in the Ross breed. In the case of the other poultry species, the RIR breed produces 42 times more AFL than duck and the quail produces 27 times more than duck. Since the 1970s, it has been proposed that AFL was merely an AFB_1_ reservoir or storage form in sensitive species like duck or rainbow trout and that this AFB_1_ reservoir could potentially lead to chronic effects because of the extension of the half-life of the toxin in the organism [3], [38], [49]. However, in a previous investigation, our research group has proposed a new role for AFL in poultry species, where it acts as a reservoir of AFB_1_ in pursuit of preventing AFB_1_ epoxidation and providing a higher tolerance to AFB_1_ exposure.

In the “cytotoxic pathway”, total production of AFB_1_ dialdehyde is the highest in quail followed by duck>RIR breed>Ross breed>turkey. The simulation for this metabolite is distributed with a peak maximum in the order quail = duck > RIR breed>Ross breed>turkey. AFB_1_ dialdehyde peak increases faster in the duck, the RIR breed, and the turkey and increases slower in the quail and the Ross breed. The simulation suggests that the cytotoxic effects in quail and duck are caused by a large AFB_1_ dialdehyde production in a short period of time. A higher concentration of AFB_1_ dialdehyde available in the hepatocyte increases the possibility of protein adduction by this metabolite. According to this, the amount of AFB_1_-Lys should be in the same way the largest in the quail and the duck, and sure enough, Fig. 4B shows that quail and duck produce the higher total amount of AFB_1_-Lys (2.8 ± 1.0 and 1.6 ± 1.2 pM respectively). At this point, the higher AFB_1_ bioactivation explains why the cytotoxic effects in quail and especially in duck are more severe than in resistant species like chicken breeds and turkey. A higher amount of available AFBO leads to a higher production of AFB_1_ dihydrodiol which in turn rearranges into AFB_1_ dialdehyde (the putative toxic metabolite of AFB_1_ associated to cytotoxic effects) to finally adduct to proteins like albumin [4].

The final step of AFB_1_ metabolism occurs when the AFAR enzyme catalyzes the reduction of AFB_1_ dialdehyde into AFB_1_ monoalcohol, and in a subsequent step, the reduction of AFB_1_ monoalcohol into AFB_1_ dialcohol. We propose to call this step for poultry species as “the elimination pathway”, which in turn can be considered as a detoxification pathway. Although the GST activity is considered a detoxification reaction because of the neutralization of AFBO, the amount of AFBO conjugated with GSH occurs only in the pM order. However, the concentrations of AFB_1_ dialdehyde, monoalcohol and dialcohol are found in nanomoles (a thousand times higher), leading to the elimination of a bulk quantity of AFB_1_ from the cell. AFB_1_ monoalcohol production in the poultry species studied, represented as the AUC value (Table 3), showed the order quail>duck>RIR breed>Ross breed>turkey. In contrast, maximum AFB_1_ dialcohol production, represented as the “b” parameter of the Hill model (Table 2) presented a reverse order (turkey>Ross and RIR breeds>duck>quail), suggesting an apparent saturation of the AFAR enzyme activity due to the overproduction of the monoalcohol in the quail but not in the turkey. Finally, the fact that all avian species reach a very close value of AFB_1_ dialcohol maximum production after 1000 minutes suggests that the AFB_1_ is eliminated in the form of dialcohol from the hepatocyte. In rats, the presence of dialcohol in urine supports this hypothesis [31]. Further, Benkerroum [5] propose that the lack of correlation between albumin adducts and AFB_1_ dialdehyde production is caused by the preferred route of reduction of the dialdehyde by AFAR enzyme into the AFB_1_ dialcohol than the adduction of AFB_1_ dialdehyde to lysine.

## 5 Concluding remarks

Information obtained from the simulation of enzyme kinetic parameters of reactions presented in Table 1 showed how the metabolism of AFB_1_ differs among the four poultry species evaluated and gives insight into the explanation of the resistance or sensitivity to AFB_1_ observed *in vivo*. It is important to highlight that the results from this study are limited to only hepatic metabolism and do not resemble the effect of AFB_1_ in extrahepatic tissues. Pursuing the explanation of tolerance and sensitivity, we focus mainly on two contrasting poultry species: the chicken and the duck. In the genotoxic pathway, it is observed in the chicken that the low production of AFBO is related to two factors: the high production of the conjugate AFB_1_-GSH and the high production of AFL. These two factors lead to a low production of DNA adducts. On the other hand, the duck presents severe signs of acute poisoning due to the high production of AFBO in a much shorter time than the other species. This is mainly related to two factors: low production of AFB_1_-GSH and low production of AFL. Therefore, the production of adducts with guanine is the highest. On the cytotoxic pathway it was observed that mid-tolerant species such as quail and turkey present extreme differences in AFB_1_-Lys production, associated to a low AFB_1_ dialdehyde elimination as AFB_1_ dialcohol in quail and a high value in turkey. This contrasting difference can explain why egg weight and egg production parameters in quail can be affected by the administration of 50 – 400 ppb of AFB_1_ [45], [46], [47], [56], meanwhile body weight in turkey is affected by the administration of 200 – 750 ppm of AFB_1_ [17], [22], [23], [34], [35], [50], [51], [64]. In the same way, the duck presents a higher AFB_1_-Lys production due to a lower AFB_1_ dialcohol production and a higher AFB_1_ dialdehyde production compared to chicken breeds.

To approach to a more precise and more comprehensive model that resembles the *in vivo* adverse effects of AFB_1_ consumption, it is necessary to investigate other parameters not considered in this study, for example, the transmembrane transport of AFB_1_ biotransformation products. For AFB_1_, it has been reported that the most probable transmembrane transport occurs by simple diffusion [41], but more recently reports has proposed the intervention of transporters of the organic anion transporters family (Organic Anion Transporter-OAT) and transporters of the organic cation transporters family (Organic Cation Transporter-OCT; [60]). Burt and Thorgeirsson [7] postulate the induction of the *MDR-1* gene (canalicular efflux transporter) as the intake route of AFB_1_ in rats, and other studies have reported that the transport of AFB_1_ and AFB_1_-GSH is mediated by the MRP1 transporter [10], [37], [59]. The study of transmembrane transport would not only allow the evaluation of *in vivo* biotransformation rates within the hepatocyte, because biotransformation rates depend on the cytosolic concentration of AFB_1_ and its biotransformation products, but also will help to find a relationship with transmembrane transporters and AFB_1_ resistance or sensitivity by removing AFB_1_ biotransformation products such as AFB_1_ dialcohol from the target cell, favoring detoxification pathways [29].

In addition to *in silico* simulation and *in vitro* assays, *ex vivo* experiments performed with cell cultures from poultry could complement *in silico* findings. It has been possible to evaluate the *in vitro* susceptibility to AFB_1_ exposure in human and mouse by using hematopoietic tissue, and in mice the *ex vivo* effects have also been investigated [52]. Another topic to consider is the adduction of AFB_1_ dialdehyde to proteins belonging to the “DNA repairing system”, which would directly affects the repair of the AFB_1_-Gua adducts produced [54]. In addition to the impact of AFB_1_ consumption on the DNA repairing system, the comparison of the effect of AFB_1_ on the chromatin condensation patterns between species would also contribute to the discovery of new factors associated with sensitivity and hepatocarcinoma development [11]. Beyond the phase I and II biotransformation processes that occur in the hepatocyte, there are no reports of phase I biotransformation reactions carried out by the CYP enzymes located in the enterocyte (the so-called “first pass” effect). For example, AFB_1_-DNA adducts have been found in rat and human enterocytes, especially in mature enterocytes expressing the CYP3A4 isoform [33]. Moreover, in Cherry Valley ducks it has been proposed that the transport processes of doxycycline hydrochloride (an antibiotic) are affected by the intake of aflatoxin B_1_ and its possible bioactivation in enterocytes [28]. In addition to enterocytes, it has also been found that blood components, such as erythrocytes, can metabolize AFB_1_ to AFL and vice versa [8]. Thus, there is a potential of biotransform AFB_1_ in tissues different from the hepatocytes, which could affect the systemic concentration of the toxin. In addition to the biotransformation processes already described in this study and in the literature, the possible glucuronidation and/or sulfoconjugation of AFB_1_ dialcohol and monoalcohol has not been explored in poultry. It has been observed that dibenzo[*a*,*l*]pyrene-trans-11,12-diol is enzymatically conjugated with glucuronic acid by human liver microsomes [48] and in intestinal cells from channel catfish (*Ictalurus punctatus*), sulfotransferase and glucuronidase activity has been found for benzo[*a*]pyrene-7,8-dihydrodiol [62]. If similar compounds to AFB_1_ can be glucuronidated and/or sulfoconjugated, the phase II biotransformation of AFB_1_ dialcohol and monoalcohol appears as a potential topic to investigate.

## CRediT authorship contribution statement

**Gonzalo J. Diaz**:Writing – review & editing, Writing – original draft. **Hansen W. Murcia**:Writing – review & editing, Writing – original draft, Methodology, Investigation, Funding acquisition, Formal analysis, Data curation, Conceptualization. **Rubén D. Acosta**:Writing – review & editing, Writing – original draft, Software, Methodology.

## Declaration of Competing Interest

The authors declare that they have no known competing financial interests or personal relationships that could have appeared to influence the work reported in this paper.

## Data Availability

Data will be made available on request.
