# Supplementary material for: In silico approximation to aflatoxin B1 metabolism and sensitivity in commercial poultry species based on empirical mathematical equations
Source: Toxicol Rep. 2024 Sep 27;13:101752. doi: 10.1016/j.toxrep.2024.101752 (PMC11483326; doi:10.1016/j.toxrep.2024.101752)
Supplement: Supplementary file 1 — Supplementary material. [file mmc1.docx]

**Supplementary material**

When the Rational model adjusts to a Gaussian curve, the time at which the C_m_ maximum (C_max_) is achieved at the “time of peak” (t_max_) which corresponds to an inflection point of the model. It can be determined by the ordinary differential equation (ODE) equaled to zero. Equation S1 presents the ODE function of the Rational model and equation S2 is obtained by equaling the ODE to cero and solving for time. At this time the C_max_ is achieved.

$\frac{d}{dx}\left( \frac{a+b*time}{1+c*time+d*{time}^{2}} \right)=\frac{{-b*time}^{2}d-2axd+b-ac}{\left( 1+cx+{time}^{2}d \right)^{2}}$ (S1)

${time}_{max}=-\frac{ad-\sqrt{d\left( a^{2}d+b\left( b-ac \right) \right)}}{bd}; -bh\neq0$ (S2)

An initial condition or initial value problem, to give an example, is the production of aflatoxin B_1_ dialdehyde by Ross breed, individual 1, whose Rational model parameters are a: -0.0565, b: 0.2618., c: -0.0325 and d: 0.001170. Solving S2 equation, the t_max_ values is 29.35 minutes. Now, solving the Rational model for this individual, the C_max_ is 7.24 pM. To corroborate the inflection point, model parameters and the t_max_ values where introduced in the ODE, resulting in a value of zero.

In the same way as the Rational model, time to achieve the C_max_ requires of the Hoerl model ODE equaled to zero. Equation S3 presents the derivative function of the Hoerl model and equation S4 presents the t_max_.

$\frac{d}{dx}\left( ab^{time}{time}^{c} \right)=a\left( b^{time}\ln(b)*{time}^{c}+c*{time}^{c-1}b^{time} \right)$ (S3)

${time}_{max}=-\frac{c}{ln(b)}$ (S4)

An initial condition is the production of AFBO by Ross breed, individual 1, whose Hoerl model parameters are a: 304.2000, b: 0.7057 and c: 0.9191. The t_max_ values is 2.63 minutes. Solving the Hoerl model for this individual, the C_max_ is 295.81 pM. To corroborate the inflection point, model parameters and the t_max_ values where introduced in the ODE, resulting in a value of zero.
